# Control and Modelling of Laser Shock Peening without Coating (LSPwC) Texture of AISI 9310 Steel

**DOI:** 10.3390/ma17194776

**Published:** 2024-09-28

**Authors:** Ping Liu, Zhandiao Yang, Cenchao Xie, Fei Yang, Liucheng Zhou

**Affiliations:** 1 School of Aeronautics, Chongqing Jiaotong University, Chongqing 400074, China; liuping@cqjtu.edu.cn (P.L.);; 2 Chongqing Key Laboratory of Green Aviation Energy and Power, Chongqing Jiaotong University, Chongqing 401130, China; 3 National Key Lab of Aerospace Power System and Plasma Technology, Air Force Engineering University, Xi’an 710038, China

**Keywords:** LSPTwC, LSPwC, LST, AISI 9310 steel, surface texturing, loss of lubrication

## Abstract

LSPwC is an important development of Laser shock peening (LSP) technology, and surface texturing is an effective method to improve tribological properties. The combination of these is expected to innovate a new surface texturing technology with a strengthing effect, but no one has attempted it. In this paper, a new LSPTwC technology combining them is innovatively proposed and validated on AISI 9310 steel, which is commonly used in helicopter transmission components for surface texturing. The LSPTwC surface was studied using an optical microscope, electron microscope, energy dispersive spectrometer, and so on. The results proved that LSPTwC is an effective texturing method of AISI 9310 steel, which modulates the texture and improves the properties, such as the microhardness increased by more than 10%. A model for calculating the texture and process parameters is also constructed on a statistical basis, and a modeling method for textured surfaces is proposed. It is verified that the calculation results and the constructed model are highly consistent with the test, with a diameter deviation <3% and depth deviation <4%. The above results can provide the experimental basis, process design method, and calculation model for single-point LSPwC texturing of AISI 9310 steel parts for helicopters, which have application value.

## 1. Introduction

Loss of lubrication (LOL) in helicopter gearboxes is a potentially catastrophic failure in which gearbox components such as gears and bearings rapidly lose lubrication, leading to a deterioration in frictional performance, resulting in an increase in surface temperatures, a reduction in material properties and a high risk of structural damage and gearbox failure [1,2,3], which is a global engineering challenge that has long plagued helicopter safety [4,5]. Surface texture is one of the most effective methods to improve tribological properties under different lubrication conditions [6,7,8]. By optimizing contact stress, cleanliness, and oil film distribution [9,10,11], tribological properties under lubrication-free conditions can be significantly improved, providing a potential solution to increase the LOL capacity [12].

LSPwC is a new type of LSP technology that does not require protective coatings, in which the laser is applied directly to the surface [13], and not only induces physical and chemical effects such as ablation, impact, remelting, and heat treatment but also creates a special strengthening surface texture that can improve tribological properties [14,15]. For example, the authors of this paper [16] carried out LSPwC treatment on AISI 9310 steel, which resulted in an increase in microhardness of about 50%, a reduction in the coefficient of friction of 12%, and an increase in wear resistance of 50 to 70%. In a further study, a work-hardening layer with a depth of more than 530 μm and a residual compressive stress layer of 510 μm were obtained, and the maximum residual compressive stress introduced was more than 748.9 MPa, and a regular weave of rhombus or rectangle was detected [17]. On the other hand, Song et al. [18] investigated the residual compressive stress introduced by LSP and found that the residual stress could significantly improve the rolling contact fatigue, and Yan et al. [19] reached similar conclusions in their study of conventional shot peening and further confirmed the beneficial effects of microstructural refinement and microhardness enhancement. In addition, Hereñú, Praveen, Zhao et al. [20,21,22] improved the high peripheral fatigue life, strength, and ductility of the material by LSPwC, Yu et al. [23] also found that it had an improved effect on micromotion wear at high temperatures, and Tong et al. [24,25] in their studies on aluminum alloys found that LSPwC improved the cavitation resistance and was further found to have the effect of improving surface wettability and pitting resistance. In conclusion, LSPwC is a surface-strengthening technique with obvious advantages and comprehensive effects, which can improve surface properties and prepare surface textures.

Although existing studies have identified the beneficial effects of LSPwC in improving tribological properties, high-temperature performance, and achieving surface texturization [16,17,18], there is no research precedent for controllable and designable texturing using LSPwC. Since material tribological properties, high-temperature performance, and fatigue strength are important factors affecting the loss of lubrication capability [11,26], it is highly expected that the application of LSPwC to surface texturing by combining the beneficial findings of existing studies will result in a novel surface texturization solution that significantly enhances the loss of lubrication capability of helicopters. Therefore, in this paper, a designable and controllable laser surface texturing (LST) based on LSPwC (LSPTwC for short) is innovatively proposed for the first time, and AISI 9310 steel, which is commonly used for helicopter transmission components, is used as the research object for the LSPTwC study, in order to validate the feasibility of the LSPTwC for helicopter transmission components and to provide experimental bases, design methodologies, and computational models for the regulation and design of the texturing structure.

## 2. Materials and Methods

### 2.1. Materials

**Figure 1:**
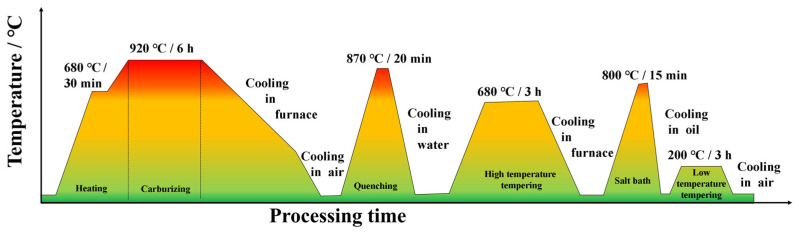
AISI 9310 steel carburizing process.

AISI 9310 steel is a carburized bearing steel with excellent properties, which is one of the most commonly used materials for the drive train of aerospace equipment [27] and is widely used in the manufacture of helicopter transmission gears, bearings, and shafts [28]. In this paper, the selected raw material is a 300 mm diameter bar; its main constituents (mass fractions) are shown in Table 1 by measured, which is cut into 5 mm slices by AQ750L wire electric discharge machine (Sodick, Suzhou, China), set electrode wire diameter 0.2 mm, voltage 15 N, feed rate 200 mm/s, and then vacuum carburizing treatment using a RX3-20-12 high-temperature box resistance furnace (HengLi, Danyang, China). The carburizing process is as follows: preheating at 680 °C, carburizing at 920 °C, carburizing period of carbon potential of 1.3%, and then slow cooling in the furnace to 350 °C air cooling; and then heating to 870 °C water cooled quenching, and then slow cooling in the furnace for tempering at 680 °C, 800 °C salt bath oil cooling, and finally 200 ℃ air cooling tempering. The detailed process is shown in Figure 1. After carburisation, remeasured under the same test conditions, the main components were as shown in Table 2, the C content on the surface of the material increased to 5.2%.

### 2.2. LSPwC Scheme

**Figure 2:**
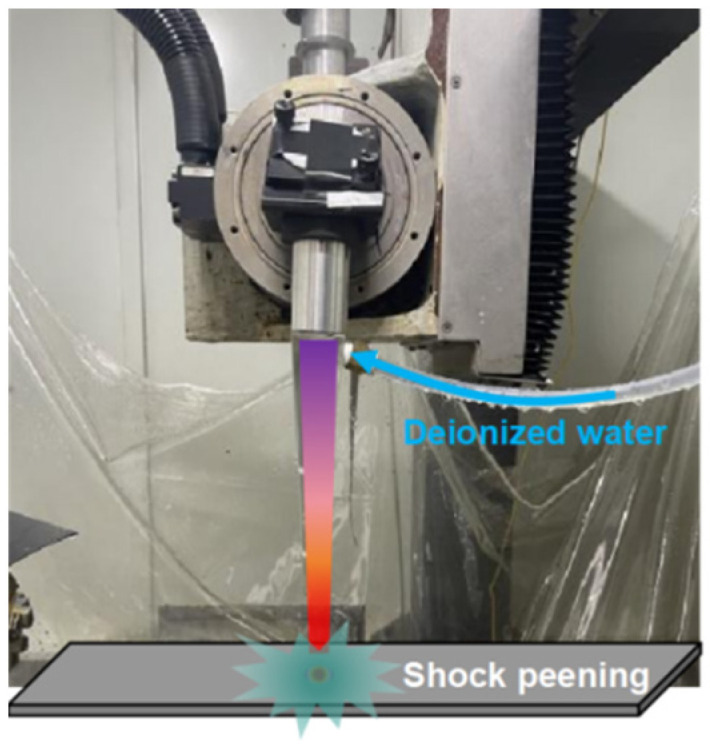
Water-optical coaxial LSP equipment.

**Figure 3:**
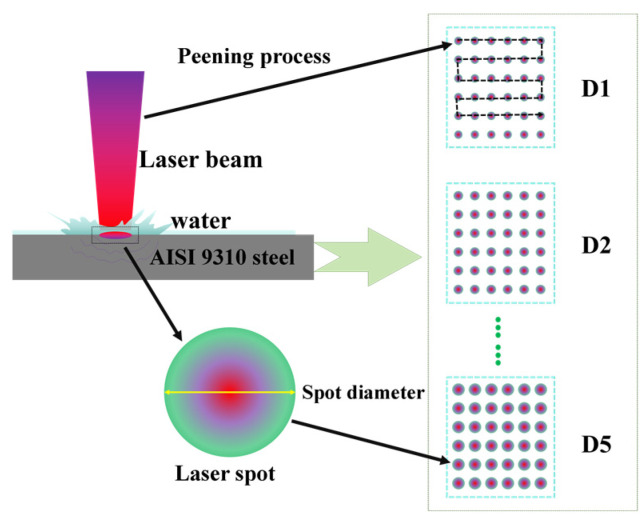
LSPTwC schemes with different spot diameters.

The test was carried out using a YSM2000-C30A water-optical coaxial LSP device (Tyrida, Xi’an, China), as shown in Figure 2. The laser is a TABOR-200 Nd:YAG laser (Joram, Beijing, China). with a pulse width of 10 ns, a circular spot shape, a maximum pulse energy of 200 mJ, a wavelength of 532 nm, a divergence angle of <2 mrad, and a repetition frequency of 50 Hz. The amplification test program employed single-point amplification, as shown in Figure 3, and the control parameters included laser energy, spot diameter, and array spacing. The detailed process parameters are shown in Table 3, and the control parameters include laser energy, spot diameter, array spacing, etc. The detailed process parameters are shown in Table 3.

### 2.3. Surface Observation and Measurement Scheme

**Figure 4:**
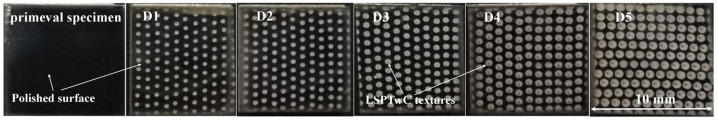
Sliced specimen of LSPTwC with different spot diameters.

Different from the traditional laser texturing technology, LSPwC is the direct action of the laser on the material surface, which not only induces plasma shock waves and forms the shock effect [29], but also directly occurs the energy transfer, resulting in ablation, remelting, and thermal impact on the material surface [30,31], resulting in the formation of multi-dimensional and multi-scale complex texture structures on the material surface. Therefore, through experimental research, obtaining the influence law of different spot diameters on the surface texture of AISI 9310 rigid under the condition of single-point LSPTwC and establishing the mapping relationship and calculation model between the laser diameter and the parameters of LSPTwC texture structure is a prerequisite for realizing the precise control of the LSPTwC surface texture structure design and peening process. Therefore, the research program of this paper is as follows:

Firstly, specimen preparation: the LSPTwC specimen and the untreated specimen were cut into 10 mm × 10 mm specimens for observation using a AP250Ls wire electric discharge machine (Sodick, Suzhou, China), set electrode wire diameter 0.08 mm, voltage 10 N, feed rate 50 mm/s, as shown in Figure 4, There were 3 specimens in each group LSPTwC parameter, to ensure that each set of data for all tests is not less than 3.

Secondly, surface 2D characterization: the surface of the specimen LSPTwC and untreated were observed using a Axiocam 208 optical microscope (Zeiss, Suzhou, China) and an G4 UX electron microscope (Helios, Davisburg, MI, USA). The parameters of SEM were set as an HV of 5.00kV, det ETD, mode SE, and WD 4.3 mm. The surface chemical elemental analysis was carried out using an Ultim Max65 EDS (Ultim, Oxford, UK) with the accelerating voltage set to 20KV and a working distance of 4 mm. The metallographic organization was observed using a ZEISS-Axiocam 208 optical microscope, with the cut surfaces of the specimens were polished using 400 mesh, 800 mesh, 1000 mesh, 1500 mesh, and 2000 mesh sandpaper in that order, and then the cut surfaces were further polished using SiO2 polish, and then corroded using 4% ethanol nitric acid solution for 10 s. The microhardness in the depth direction of the cut surface was measured by the EM500-3A microhardness tester (HengYi, Shanghai, China) set to a load of 300 g, time 10 s, and one group was collected at 10 μm intervals from the surface to 100 μm, one group was collected at 50 μm intervals from 100 μm to 500 μm, one group was collected at 100 μm intervals from 500 μm to 1000 μm.

Finally, Surface 3D characterization: thirdly, a GT-K optical profiler (Bruker, Billerica, MA, USA) and a Bruker Dimension ICON atomic force microscope (Bruker, Billerica, MA, USA) were used to scan the surface profiles untreated and LSPTwC specimen to characterize the 3D profiles, sampling intervals are 0.1 μm and 1 nm. Through multi-dimensional and multi-scale observation and characterization, we provide the experimental basis and data support for analyzing the influence of spot diameter on the LSPTwC texture structure of AISI 9310 steel material and establishing the relationship model between spot diameter and texture structure.

## 3. Result and Discussion

### 3.1. 2D Texture Observation and Analysis

#### 3.1.1. Optical Microscope Observations and Analysis

**Figure 5:**
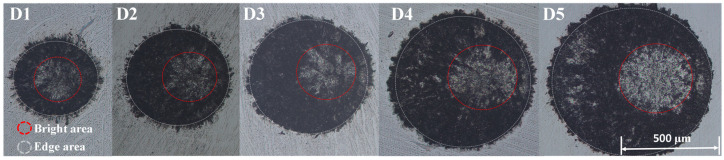
Overall view of LSPTwC surface with different spot diameters (OM 10×).

Different spot diameters of LSPTwC specimens were observed under a 10× light microscope, as shown in Figure 5. Regardless of the diameter, LSPTwC formed a well-bounded crater texture on the surface of AISI 9310 steel, and there was a clear difference in topography from the center to the edge. The center of the crater showed a bright metallic color, while the main body of the crater showed a black ablation feature, and the edge area showed an outwardly jetted extended texture. The highlighted area is not at the geometric center of the crater but is eccentric within the scan line, and the distance to the rim increases only slightly with increasing diameter. For the same crater, the further away from the highlighted area, the clearer the edge and the less jet-like the texture. The overall surface morphology of the crater indicates that ablation, melting, jetting, and remelting deposition occurred on the surface of the AISI 9310 steel during the LSPTwC process. The size of the highlighted area and the eccentricity of the crater increase with increasing spot diameter, suggesting that the change in spot diameter affects the laser energy distribution and its effect on the material surface.

**Figure 6:**
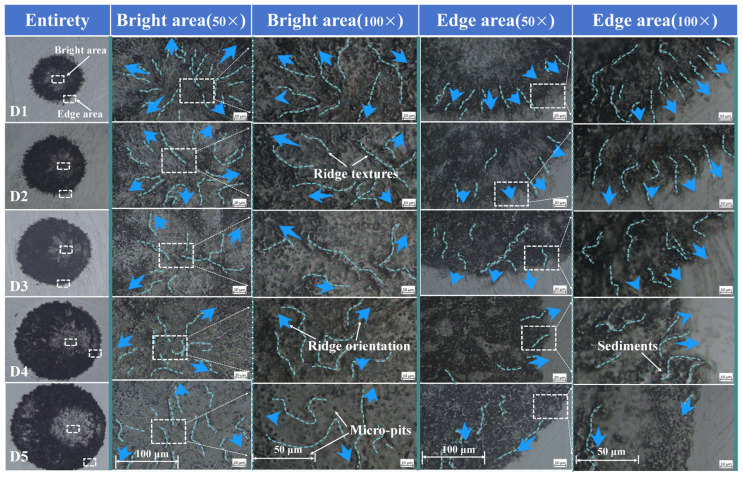
Comparison of LSPTwC surfaces with different spot diameter (multi-scale OM).

To further clarify the effect of spot diameter on the crater’s microtexture structure, the microscope’s resolution was adjusted to 50× and 100×, and the center and edge regions of the crater were compared, as shown in Figure 6. Jet-like textures are commonly found in the interior of the crater, and the smaller the diameter, the longer and thinner the jet-like textures are, and the more pronounced the outward jet feature is. All jet textures originate from the highlighted regions and show outward extension. The jet-like texture at the edge becomes shorter and shorter as the spot diameter increases, and remelting accumulation is found at the edge of the 650 um diameter crater. The distal edge of the 750 um diameter crater does not even have a jet-like texture, which is only characterized by ablation and remelting. In contrast, a wave-like remelting accumulation is found in the inner region near the center. The above texture and structural features are obviously the result of deposition and stacking of ablative and molten materials formed by splashing and flowing under the shock wave’s action, and the shock’s source is located in the highlight area. Therefore, the central area has less ablative and re-melted material deposited, giving it a metallic sheen, while the surrounding area has a large amount of ablative and re-melted material deposited, giving it a black ablative feature. In addition to the jet and wave textures, the surface of LSPTwC shows a large number of micro-nanometre-sized circular and reticulated pits under a high magnification microscope, with intact edges of the micro-pits and black bottoms and depositional structures in the centers of some of the micro-pits. Crater edges can be observed as heat-affected zones, and a large number of remelted depositional structures can be seen under a high-power magnification microscope. The remelted depositional structures decrease in diameter but increase in size. Large remelted deposits will show a slag structure. Combining the results of the optical characterization with different resolutions, it is easy to see that the impact effect is weakened as the spot diameter increases, the ablation and remelt residues increase, the ablation and remelt depositional structures increase, and the microtexture structure of the crater rim deteriorates.

#### 3.1.2. Electron Microscope Observations and Analysis

**Figure 7:**
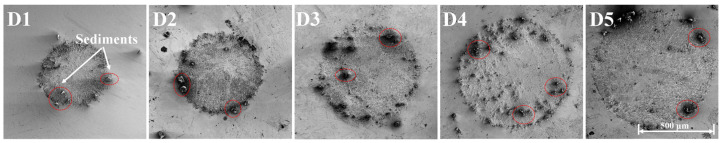
Overall of LSPTwC surface with different spot diameter (SEM mag300×).

The observation and characterization of LSPTwC crater by scanning electron microscope (SEM) can avoid unfavorable interferences such as focal length and color of an optical microscope. Firstly, the SEM mag was adjusted to 300× for observation and characterization, and the results are shown in Figure 7. The center region of all craters is flatter than the edge region, and the texture structure is more delicate. A large amount of remelted deposition texture exists at the edge of craters, and even large aggregated remelted deposition material is found. The aggregated remelted deposits and their surrounding areas show distinct brightness variations, with a significant increase in the brightness of the deposits themselves and a significant decrease in the brightness of the surrounding areas. The brightness variation reflects the different conductivity of different regions of the crater, indicating that LSPTwC has caused complex physical and chemical changes on the surface of the AISI 9310 steel, resulting in surface modification of the material. The distribution of the re-melted deposits was significantly correlated with the crater diameter—the larger the diameter, the more deposits, the larger and closer to the center. However, when the diameter reached 650 μm, the phenomenon showed an improving trend.

**Figure 8:**
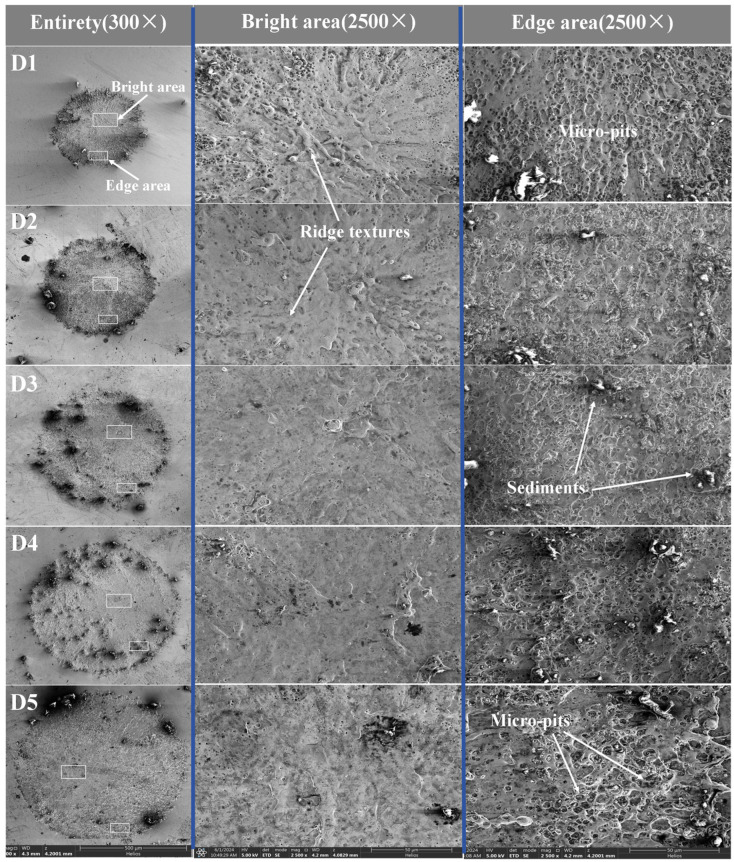
Comparison of LSPTwC surfaces with different spot diameter (multi-scale SEM).

The mag was then adjusted to 2500×, and the center and edge regions of the crater were locally enlarged and the characterization results are shown in Figure 8. It can be seen that the overall morphology of the crater center tends to become more straightforward with the increase of the spot diameter, and the texture and micropores decrease and become smaller. In contrast, the remelted deposit structures at the crater edge increase with the increase of the spot diameter, the micropores increase and become larger, the edges of the micropores gradually become indistinct, and even the cross-over phenomenon occurs. The loose deposit structures inside the micropores gradually increase and become larger. The large re-melted deposits showed welding and adhesion under 2500×, which were the adherent materials produced by splash dropping.

**Figure 9:**
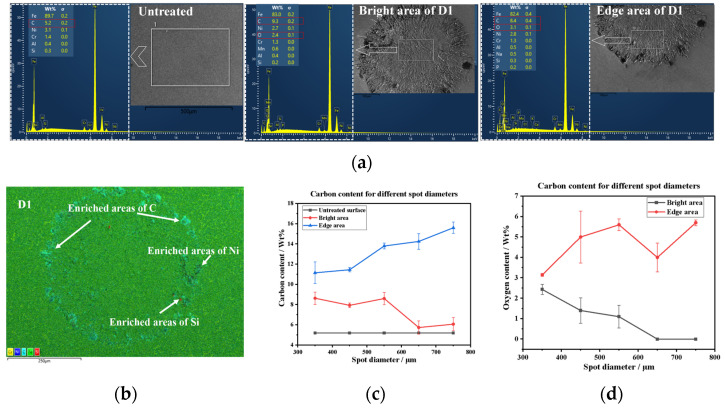
Analysis of chemical elements with different parameters and locations: (**a**) EDS testing; (**b**) EDS scanning; (**c**) carbon content analysis; (**d**) oxygen content analysis.

Comparing the LSPTwC and untreated specimens, carbon and oxygen elements are the two components with the most significant changes, and the discovery of oxygen indicates the presence of oxidation phenomena such as ablation during the LSPTwC, and the increase in carbon has been similarly found in the existing studies as a change in favor of dry friction self-lubrication [16,17]. Comparing the same LSPTwC texture, the bright area and the edge area are typically different areas, and the carbon and oxygen content of the edge area is significantly higher than that of the bright area, indicating that remelted deposition enhances the change in the chemical element content, which is also evidenced by the enrichment of other elements in the remelted deposition texture. The edge area is relatively protruding, and its higher carbon and alloying elements content will be favorable for improving tribological properties [32,33]. The variation of the chemical element contents of the bright area and edge area with spot diameter is shown in Figure 9a,b; with the increase of spot diameter, the carbon content of edge area increases, the carbon content of bright area decreases, the oxygen content of edge area increases and then decreases, and the oxygen content of bright area decreases continuously, as shown in Figure 9c,d. The above results show that a smaller spot diameter can obtain a more uniform chemical composition of the texture, while the carbon and oxygen content of the bright area above 650 μm diameter is basically the same as that of the untreated samples, which indicates that LSPTwC has almost no modulation effect on the chemical composition under this condition.

**Figure 10:**
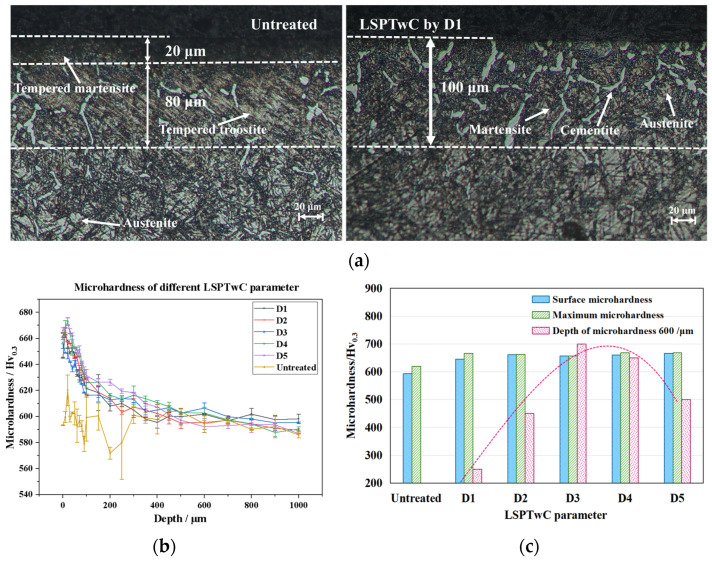
Metallographic and microhardness analyses. (**a**) Metallographic analysis of untreated and D1, (**b**) microhardness at different depths, and (**c**) maximum microhardness and hardening depth.

The metallographic corrosion characterization results comparing the LSPTwC of D1 and untreated specimens are shown in Figure 10. The metallographic phase of untreated specimens is a layered structure consistent with the heat treatment process. After LSPTwC, the original layered structure was changed, forming a continuous gradient structure from the matrix to the surface grains, which were gradually refined, as shown in Figure 10a, a typical LSP strengthening phenomenon [34]. The high-temperature tempering organization of the subsurface layer was transformed into martensite, reflecting the quenching effect of the fast heating and cooling of the laser and confined water layer. It was also found that many reticulated carburite structures precipitated at the martensitic grain boundaries, extending from the matrix to the surface, which is consistent with the elevated surface EDS carbon content. Grain refinement, martensite, and carburite all have a strengthening effect [35,36,37] in order to confirm the strengthening effect of LSPTwC on the material, the microhardness was measured as in Figure 10b, the microhardness of all specimens was elevated by more than 10%, and almost the surface is the hardest layer, which is consistent with the results of the metallurgical characterization, and the hardening depth of D3 was found to be the largest, which reached 700 μm as shown in Figure 10c, which is supposed to be a result of the combined effect of force and heat.

### 3.2. 3D Texture Observation Analysis and Modeling

#### 3.2.1. 3D Texture Observation and Analysis

**Figure 11:**
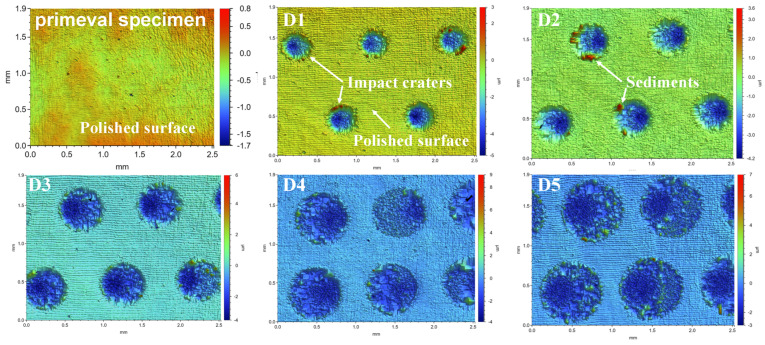
Overall LSPTwC surface with different spot diameters (OSP).

The optical profilometer characterization results provide the experimental basis and data support for us to quantitatively analyze the LSPTwC crater 3D texture structure of AISI 9310 steel. Figure 11 shows the overall 3D morphology of the original specimen and LSPTwC specimens with different spot diameters. The results show that LSPTwC produces a typical impact crater on the surface of AISI 9310 steel, and the 3D morphology of the crater textures formed by the same diameter spot has a good consistency, which almost does not affect the surface outside the impact area, except for some spatter and accumulated material at the edge.

**Figure 12:**
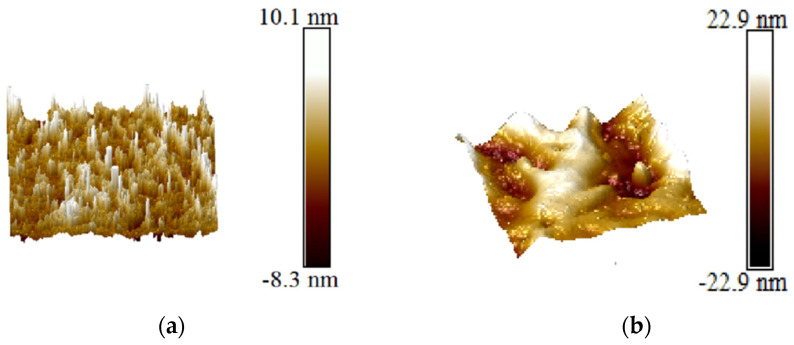
Atomic force microscopy characterization of (**a**) original polished surface and (**b**) LSPTwC crater center.

Atomic force microscopy (AFM) was further used to observe and analyze the highlighted regions observed by OM at the nanoscale, as shown in Figure 12a,b. Before LSPTwC, the surface of the pristine specimen showed a capillary columnar structure characteristic of machining, with surface undulations up to about 40 nm. After LSPTwC, the surface of the specimen formed a complete and s mooth microcrystalline surface, and the rough peaks became coarse and rounded. This indicates that the polished surface of the sample, although bright and intact at the general microscopic scale, is not intact at the nanoscale. On the other hand, the remelting effect of LSPTwC can produce very intact crystalline surfaces at the micrometer and nanometre scales.

**Figure 13:**
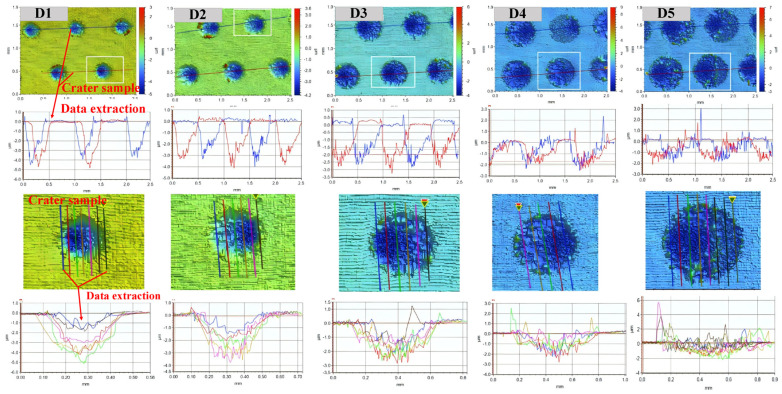
Extraction of surface profile data.

Since all craters have a symmetrical shape in the laser scanning direction, this paper extracts the crater surface profile data in the cross-section in the scanning direction and perpendicular to the cross-section in the scanning direction, using the laser scanning line as a reference and further quantitatively analyses the effect of spot diameter on the characteristic parameters of the crater surface profiles, as shown in Figure 13. The crater profile within the symmetric plane in the scanning direction is non-symmetric in shape and has a steeper distribution on one side, which is consistent with the eccentricity results observed by OM and SEM. However, the crater profile within the vertical section in the scanning direction is symmetrical. In addition, the LSPTwC texture has noisy micrometer-scale roughness peaks and valleys in addition to the crater, leading to a significant increase in surface roughness.

**Figure 14:**
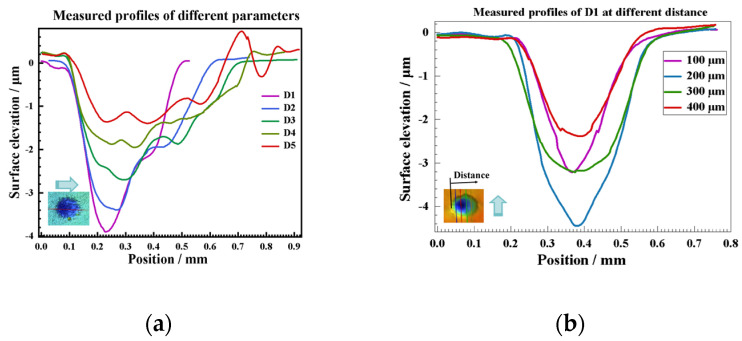
Long wavelength pass of (**a**) symmetry section and (**b**) vertical section.

**Figure 15:**
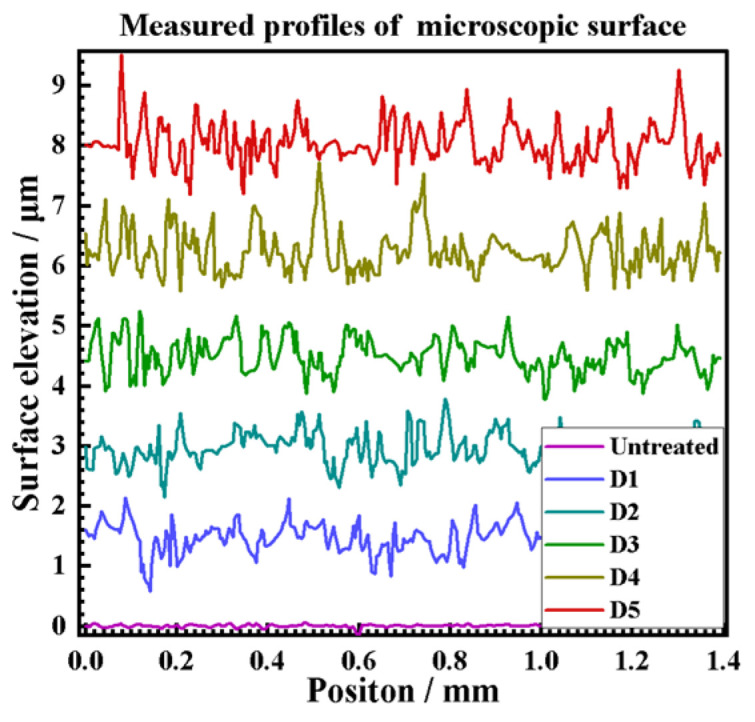
Short wavelength pass.

Obtaining crater texture structure characteristic parameters and their changing law is the key to realizing texture structure design and accurate control, but the raw surface profile data contains rough peaks and valleys, which poses challenges to crater structure characterization. Therefore, this paper adopts the Gaussian filtering method to separate the long- and short-period surface profile data, and the cutoff wavelength is set to 0.1 mm. The results of the long-wave cutoff filtering are shown in Figure 14a,b, and the results of the short-wave cutoff filtering are shown in Figure 15. The results show that with the increase of the spot diameter, the stability of the crater texture profile decreases, and the internal non-periodic fluctuating texture gradually increases. The rough peaks and valleys are basically consistent with the general rough surface shape and, thus, can be used to analyze the roughness.

According to the characteristics of the laser energy distribution and the characteristics of the measured profile curve, this paper first tries to use normal and skewed functions to fit and analyze the crater profile curve, and the selected models are the origin basic functions Gauss Equation(1) and the LogNormal Equation (2), in which y is the surface elevation, x is the calculate position, $y_{0}$ reflects the elevation of the original surface, and $x_{c}$ is related to the polar transverse coordinates, and ω and A reflect the curve width and depth, respectively.

(1) $$y=y_{0}+\frac{A}{\omega \sqrt{\pi /2}}{exp}^{-2\frac{{\left(x-x_{c}\right)}^{2}}{\omega^{2}}}$$

(2) $$y=y_{0}+\frac{A}{\sqrt{2\pi }\omega x}{exp}^{\frac{-{\left(\ln\frac{x}{x_{c}}\right)}^{2}}{2\omega^{2}}}$$

**Figure 16:**
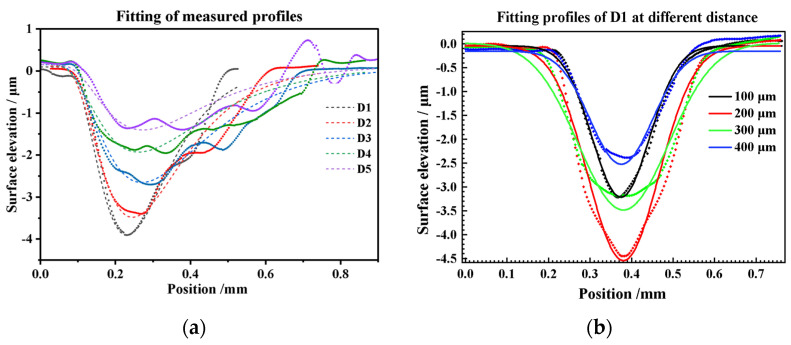

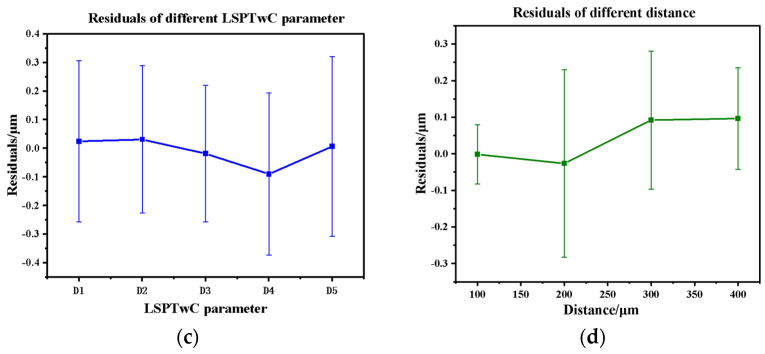
Fitting the long wavelength pass of (**a**) the symmetry section, (**b**) the vertical section profiles, (**c**) the symmetry section profiles fitting Residuals, and (**d**) the vertical section profiles fitting residuals.

The long wavelength pass data in Figure 13 are fitted by Origin2021, and the results are shown in Figure 16a,b. The LogNormal model can fit the symmetry section profiles with an average residual of less than 0.096 μm. Still, the fitting effect worsens when it is far away from the bottom or greatly increases the spot diameter. A large number of irregular textures are found in the area of optical observation, which is the result of the decrease in energy density. Gauss fits the profiles of the vertical section of D1 better; the average residual is less than 0.089 μm, as shown in Figure 16c,d. The results indicate that the morphological characteristics of the LSPTwC craters indeed match the laser energy distribution, and the eccentricity of the craters is likely to be caused by the biased distribution of the laser energy. Therefore, increasing the laser energy density can better control the crater structure; changing the spot energy distribution is an effective means to regulate the internal texture structure of the LSPTwC crater. 

**Figure 17:**
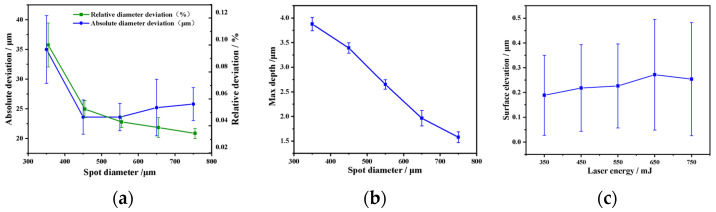
Statistical analysis of different spot diameters (**a**) diameter deviation, (**b**) max. depth, and (**c**) surface roughness.

According to the shape and change rule of crater profiles with different diameters, this paper further collected several sets of data. Statistically, it analyzed the filtered feature parameters to obtain the statistical curves of the diameter deviation and maximum depth of crater with the change of spot diameter, respectively. The results are shown in Figure 15. The diameter deviation and maximum depth of the crater decrease with the increase of the spot diameter, as shown in Figure 17a,b. By using Equation (3) to calculate the roughness and peak-valley variance, it was found that the surface roughness increased more than 10 times relative to the original surface, as shown in Figure 17c. The surface roughness of the craters with different diameters had a good consistency, which increased slightly with the increase of the spot diameter, and the peak-valley variance also increased, and the maximum roughness was obtained at a spot diameter of 650 μm, indicating that the variation of the spot diameter parameter can also regulate the roughness of the LSPTwC surface.

(3) $$Ra=\frac{1}{l}\int_{0}^{l}\;|Z(x)|dx$$

#### 3.2.2. 3D Texture Modeling and Verification

Since the spot diameter is only a limitation of the effective area of action of LSPTwC, it is difficult to reflect the intrinsic factors that affect the crater texture characteristics. Existing studies have shown that laser energy density is a key process parameter for LSP-induced shock waves [38]. Therefore, in this paper, we consider the energy density as the core parameter to replace the spot diameter as a reference for analysis, and its interconversion relationship is shown in Equation (4). Where *E_d_* is the laser energy density, *E* is the laser energy, and *d* is the spot diameter.

(4) $$E_{d}=\frac{E}{\pi d^{2}/4}$$

**Figure 18:**
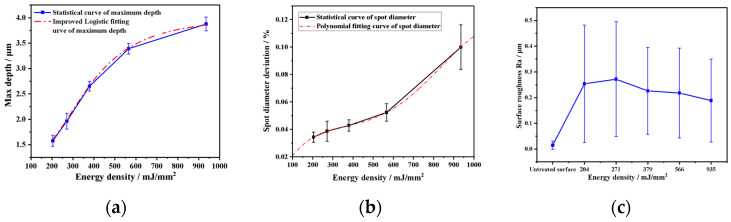
Statistical analysis of different spot diameters with laser energy density: (**a**) diameter deviation, (**b**) max. depth, and (**c**) surface roughness.

The energy densities of LSPTwC of different diameters were calculated using Equation (2), as shown in Table 4:

Taking the laser energy density as the reference value, the relative deviation of crater diameter, maximum depth, and surface roughness is analyzed again, and the results are shown in Figure 18, and the statistical curves of the relative deviation of crater diameter, maximum depth and surface roughness with respect to the laser energy density are obtained. The maximum depth of the crater increases with the increase of the laser energy density, but the trend of increase is significantly slowed down and gradually approaches the limit value after 550 (mJ/mm^2^), as shown in Figure 18a. After 550 (mJ/mm^2^), the increase slows down significantly and gradually approaches the limit. The relative deviation of the spot diameter increases with increasing laser energy density and shows an accelerating trend, as shown in Figure 18b. The surface roughness and the variance of the roughness front valley have extreme values, and when the energy density deviates from the extreme value, the surface roughness will be improved, as shown in Figure 18c.

Further observation of the change rule of crater profile characteristic parameter with laser energy density shows that there is a significant correlation between the two, which creates the conditions for establishing the relationship model between crater profile characteristic parameter and laser energy density. For this purpose, according to the basic law that the shape of the curve and the LSP affect the amount of plastic deformation of the material [39], the improved logistic Equation (7) and the multinomial Equation (8) were used in this paper to fit the maximum depth and the spot diameter deviation. Among them, the improved logistic model was optimized based on the standard logistic Equation (5).

(5) $$y=\frac{A}{1+{exp}^{-k\left(x-x_{0}\right)}}$$

where *A* is the maximum saturation, *k* is represents the growth rate of the curve, *x*_0_ is the centre position of the curve, and exp is the base of the natural logarithm.

Considering that the capacity of the equipment limits the laser energy in practical applications, a maximum and minimum value limit always exists, so the model is adjusted accordingly. Specifically, the standard logistic model is improved to Equation (6) by introducing non-zero maximum and minimum parameters *A*_1_ and *A*_2_.

(6) $$y=A_{2}+\frac{A_{1}-A_{2}}{1+e^{-k\left(x-x_{0}\right)}}$$

The relative exponential term was improved to ${\left(x/x_{0}\right)}^{p}$ to obtain more flexibility to get the final improved logistic Equation (7).

(7) $$y=A_{2}+\frac{A_{1}-A_{2}}{1+{\left(x/x_{0}\right)}^{p}}$$

The Multinomial Equation (8) is a standard polynomial model, where y is the relative deviation of the diameter, $y_{0}$ is the static relative deviation of the diameter, *x* is the laser energy density, and $A_{i}$ is the weight of the different exponential phases of the laser energy.

(8) $$y=y_{0}+\sum_{1}^{n}A_{i}\times x^{i}$$

The fitting results are shown in Figure 16a,b, and both models have good fitting results. Plug in Equation (4), the spot diameter–maximum depth Equation (9) and spot diameter-diameter relative deviation Equation (10) were obtained from the fitting.

(9) $$y=\frac{1.2-3.9}{1+{\left((\frac{P}{\pi d^{2}/4})/340\right)}^{3.5}}+3.9$$

(10) $$y=3.495\times 10^{-4}\times (\frac{P}{\pi d^{2}/4})-1.007\times 10^{-6}\times {\left(\frac{P}{\pi d^{2}/4}\right)}^{2}+1.313\times 10^{-9}\times {\left(\frac{P}{\pi d^{2}/4}\right)}^{3}\;-5.424\times 10^{-13}\times {\left(\frac{P}{\pi d^{2}/4}\right)}^{4}-0.052$$

From the laser energy density calculation Equation (4), it can be seen that the energy density change is inversely proportional to the square of the diameter. Therefore, in this paper, the highest spot energy density position is taken as the benchmark, and the energy distribution is corrected according to the square of the diameter change to construct the crater profile coordinate calculation Equation(11). The profile depth of the crater is then scaled equally to the maximum profile depth to construct the profile depth calculation Equation (12).

(11) $$\left\{\begin{array}{c} x=x_{c}-{\left(\frac{x_{c}}{d_{cn}}\right)}^{2}\times \Delta d_{cn}\;x_{c}<0\\ x=x_{c}+{\left(\frac{x_{c}}{d_{cp}}\right)}^{2}\times \Delta d_{cp}\;x_{c}>0 \end{array}\right.$$

(12) $$y=\frac{d_{max}}{d_{cmax}}\times d_{c}$$

**Figure 19:**
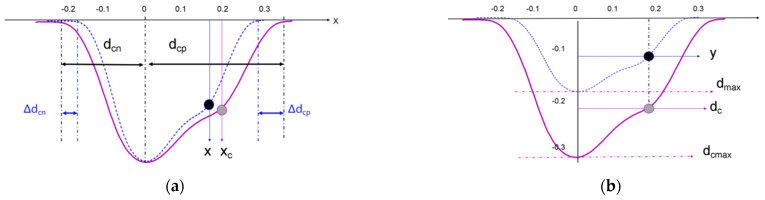
Parameter definition schematic (**a**) coordinates, (**b**) depth.

The parameters are defined as shown in Figure 19. *x* is the computed profile coordinate, *x_c_* is the calibrated profile coordinate of the computed point,$\;d_{Cn}$ is the total length of the calibrated profile for $x_{c}<0$, $\Delta d_{c}$ is the increment of the profile computation in the direction of $x_{c}<0$, $d_{Cp}$ is the total length of the calibrated profile for $x_{c}>0$, $\Delta d_{p}$ is the increment of the profile computation in the direction of $x_{c}>0$, which is defined in Figure 19a, and the increment of profile computation is corrected by Equation (8) in model (12), where y is the profile calculation depth, $d_{max}$ is the maximum depth of the profile obtained by calculation, $d_{cmax}$ is the maximum depth of the profile obtained by calibration, and $d_{c}$ is the profile depth of the calibration point at the calculation point, which is defined in Figure 19b.

**Figure 20:**
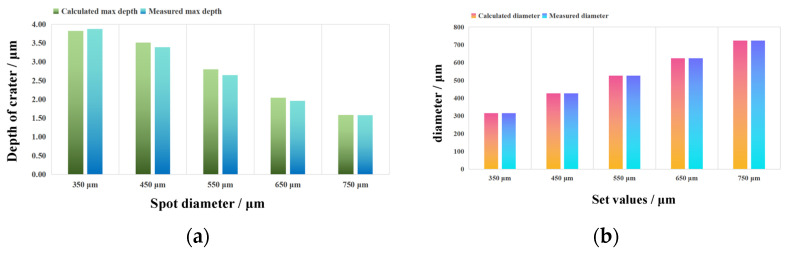
Calculated LSPTwC texture feature parameters with different spot diameters: (**a**) max. depth and (**b**) spot diameter.

In order to verify the accuracy of the above models, the spot diameter–maximum depth Equation (9) was used to calculate the maximum depths of craters with different diameters and compared with the measured statistical results; the results are shown in Figure 20a. The comparison shows that the average inaccuracies with the statistical mean is 3.08%, and the maximum inaccuracies is 5.79%, which is within the range of statistical variance, indicating that the model can accurately calculate the maximum depths of craters with different spot diameters. The spot diameter–diameter relative deviation Equation (10) was used to calculate the crater diameter for different spot diameters and compare it with the measured statistical results shown in Figure 20b. The average inaccuracies are 0.03%, the maximum inaccuracies are 0.07% from the statistical mean, and the corrected crater diameter has a deviation of less than 1 μm from the statistical mean, which is much smaller than the statistical variance, indicating that the model can be used to accurately calculate the crater diameter.

**Figure 21:**
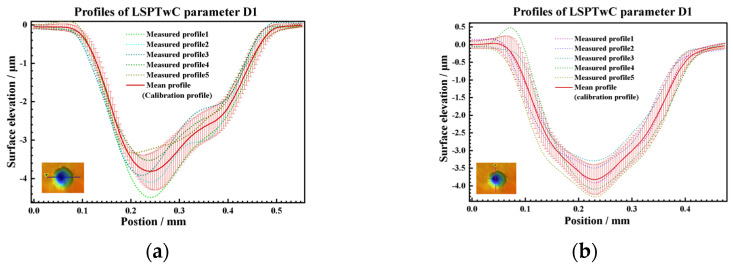
Calibration profiles data of (**a**) symmetrical section and (**b**) vertical section.

**Figure 22:**
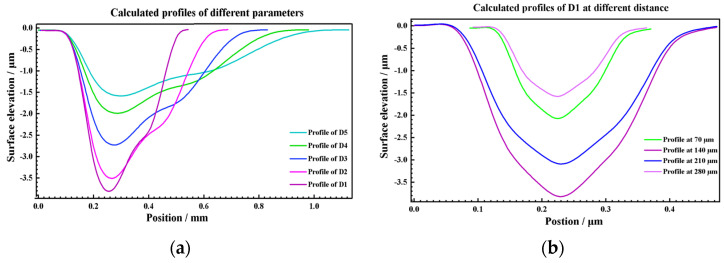
Calculated profile data of (**a**) symmetrical section and (**b**) vertical section.

**Figure 23:**
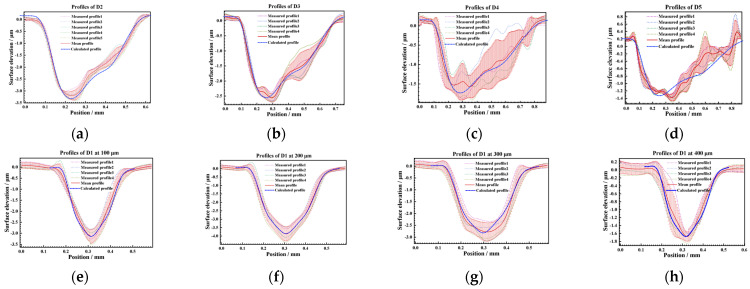
Comparison of measured and calculated profiles: (**a**–**d**) symmetrical section and (**e**–**h**) vertical section.

To make the calculation more accurate, the 350 μm peening crater with the best surface integrity was selected as the calibration base in this paper. Several groups of 350 μm crater profile data were collected for statistics, and the mean curve was obtained as calibration data, as shown in Figure 19. Based on the profile coordinate calculation Equation (11) and the profile depth calculation Equation (12), the profiles of 450 μm, 550 μm, 650 μm, and 750 μm were jointly calculated on the basis of the calibration data, as shown in Figure 21. By comparing the average value of the measured profile, the calculated profile has good consistency, as shown in Figure 22. In addition, under the condition of low energy density, the measured profiles also show irregular fluctuations of the internal profiles of the crater, which is consistent with the OM and SEM characterization results, as shown in Figure 23, the higher the energy, the better the consistency, that indicates that maintaining a higher energy density is conducive to obtaining more regular and controllable crater textures.

In conclusion, the calculation method of arbitrary laser spot diameter LSPTwC texture is as follows: 

(1) The maximum depth and diameter are calibrated according to the test data. 

(2) According to the laser energy, the maximum depth and coordinate correction are calculated using Equations (9) and (10). 

(3) Calculate 2D profile data according to Equations (11) and (12). 

(4) Use the same method to calibrate the reference profile in the vertical plane, then calculate the profile’s maximum depth in the vertical plane, coordinate correction, and other characteristic parameters according to the profile data in the symmetrical plane.

(5) Finally, Equations (9) and (10) were used to calculate all the profile data in the vertical plane, and an LSPTwC textured 3D surface was constructed.

**Figure 24:**
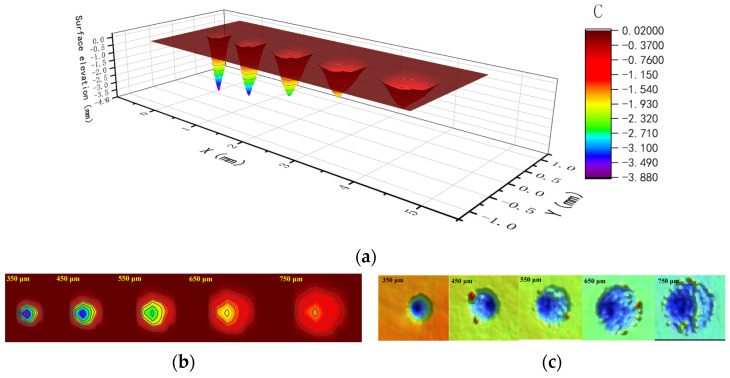
Comparison of calculated and measured textures of different diameters: (**a**) calculated 3D textures, (**b**) calculated 2D textures, and (**c**) measured 3D textures of overlooking.

Through the above methods, using the 350 μm crater as the calibration data, LSPTwC crater textures of 450 μm, 550 μm, 650 μm, and 750 μm were successfully constructed, as shown in Figure 24. Compared with the experimental results, the calculated texture structures not only have the same main features but also can eliminate the texture defects of remelting accumulation. They can also be used for the design of LSPTwC texture structures and the calculation of process parameters. 

**Figure 25:**
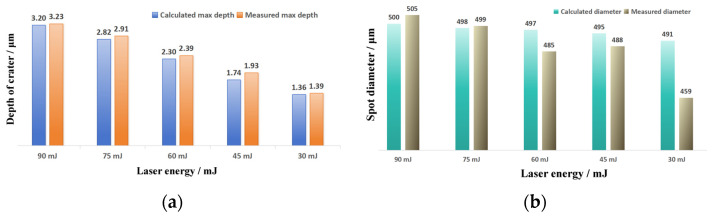
Calculation of the LSPTwC texture feature parameters with different laser energies: (**a**) max. depth and (**b**) spot diameter.

**Figure 26:**
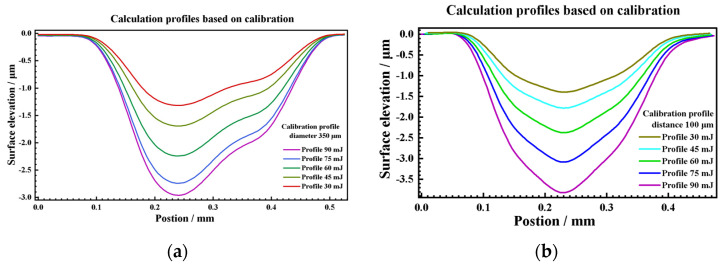
Calculated profiles data of (**a**) symmetrical section, (**b**) vertical section.

**Figure 27:**
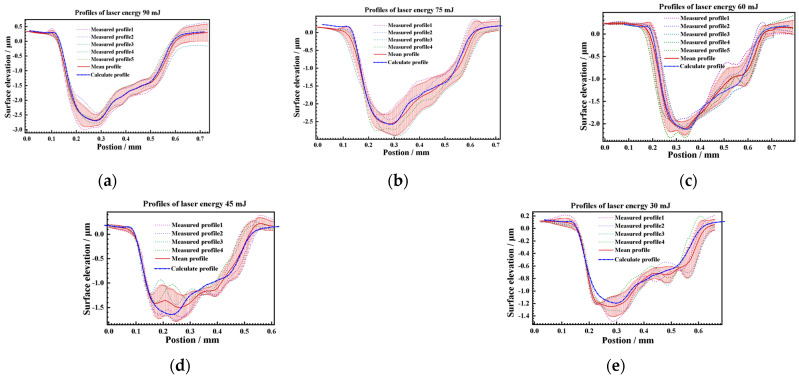
Comparison of calculated and measured profiles of different energies as. (**a**) 90 mJ, (**b**) 75 mJ, (**c**) 60 mJ, (**d**) 45 mJ, and (**e**) 30 mJ at symmetrical section.

**Figure 28:**
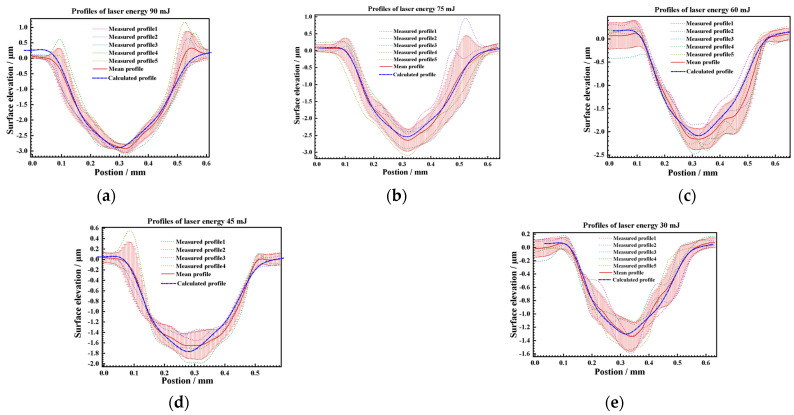
Comparison of calculated and measured profiles of different energies as. (**a**) 90 mJ, (**b**) 75 mJ, (**c**) 60 mJ, (**d**) 45 mJ, and (**e**) 30 mJ at vertical section.

Further attempts were made to construct texture models of 90 mJ, 75 mJ, 60 mJ, 45 mJ, and 30 mJ peening craters with a 500 μm spot. The maximum depth and diameter are shown in Figure 25. The average deviation of the maximum depth of the crater is 3.94%, and the maximum deviation is 9.67%, which is within the range of statistical variance. The average diameter deviation of the crater was 9 μm, which is less than the diameter calibration accuracy of the LSP instrument. The maximum deviation was 32 μm, which was less than the statistical variance. Then, the profiles of 90 mJ, 75 mJ, 60 mJ, 45 mJ, and 30 mJ under the condition of a 500 μm spot were calculated using the above profile coordinate calculation Equation (8) and the profile depth calculation Equation (9) as Figure 26. The symmetric section profiles of the craters with different energies are shown in Figure 27. Compared with the measured statistical data, the calculated profiles are in good agreement with the measured profiles. The vertical profile of the craters at different energies is shown in Figure 28. Compared with the measured statistical data, the calculated profiles also agree with the measured profile.

**Figure 29:**
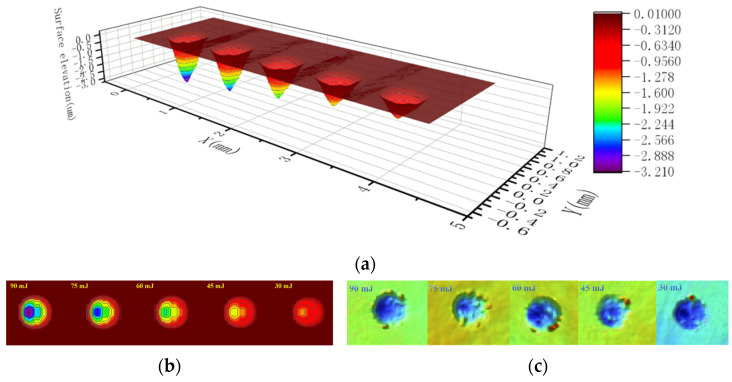
Comparison of calculated and measured textures of different energies: (**a**) calculated 3D textures, (**b**) calculated 2D textures, and (**c**) measured 3D textures of overlooking.

As shown in Figure 29, the LSPTwC crater textures of 90 mJ, 75 mJ, 60 mJ, 45 mJ, and 30 mJ were successfully constructed, as shown in Figure 29a. The shape and elevation distribution of LSPTwC textures with different energies calculated and constructed by this method still agree with the measured results, as shown in Figure 29b,c.

In summary, the above method can satisfy the calculation of spot diameter and energy regulation at the same time and can accurately design the surface texture of LSPTwC with different spot diameters and different laser energy, which can provide a reliable calculation method and model for the surface texture design, simulation, and process parameter calculation of a single-point LSPTwC scheme.

## 4. Conclusions

In this paper, the first LSPTwC scheme using LSPwC to achieve controllable and designable LST is proposed, and an LSPTwC study is carried out on AISI 9310 steel, which is commonly used in helicopter transmission components in response to the need for LOL-resistant texture on the surface of helicopter transmission components. The study not only verified the feasibility of LSPTwC and proved the combined force and thermal strengthening effect of LSPTwC but also proposed the LSPTwC formation modeling method, constructed the calculation model, and carried out experimental verification; this has never been done before [16,17]. The main conclusions of the whole study are as follows:

(1) The spot diameter, energy density, and distribution can precisely control the macro-structural parameters of the LSPTwC crater texture and the micro-texture structure inside the crater. Therefore, LSPwC can be fully applied to controllable and designable laser surface texturing, i.e., the innovative LSPTwC technology proposed in this paper is feasible.
(2) LSPTwC also has both physical and chemical effects, such as ablation, impact, remelting, and heat treatment, which can change the chemical composition, microstructure, metallographic structure, etc., of the surface material, thus forming a strengthening effect. For AISI 9310 steel, LSPTwC increases the microhardness by more than 10% with only 9.6% coverage and produces a gradient structure and a reticulated carburic organization that extends to the surface, demonstrating the technological advantages of texturizing and strengthening.
(3) Based on the fitting of the experimental data, the characteristic parameters of the LSPTwC crater texture can be identified more accurately, and a higher-accuracy computational model can be constructed. Based on the calibrated profile curves with higher energy density, the textured surface models with different LSPTwC parameters can be constructed more accurately with a diameter deviation of <3% and a depth deviation of <4%. The above results can provide a design method and calculation model for single-point LSPwC crater texturing and its process parameter design of AISI 9310 steel parts for helicopters, which has engineering value.

## Figures and Tables

**Table 1:** Main chemical elements and contents of AISI 9310 steel.

C	Mn	Si	Cr	Ni	Mo	Al	O	Fe
0.1	0.6	0.2	1.3	2.7	0.3	0.4	0	94.88

**Table 2:** Main chemical elements and contents of AISI 9310 steel after carburization.

C	Mn	Si	Cr	Ni	Mo	Al	O	Fe
5.2	0.6	0.3	1.3	3.1	0.3	0.4	0	89.7

**Table 3:** Different spot diameters LSPTwC parameter list.

Parameter ID	Spot Diameter (μm)	Cols Spacing (μm)	Line Spacing (μm)	Laser Energy (mJ)
D1	350	1000	1000	90
D2	450	1000	1000	90
D3	550	1000	1000	90
D4	650	1000	1000	90
D5	750	1000	1000	90

**Table 4:** Calculated laser energy density (laser pulse energy 90 mJ).

Spot Diameter (μm)	350	450	550	650	750
Energy Density (mJ/mm^2^)	936	566	379	271	204

## Data Availability

The data used to support the findings of this study are available from the corresponding author upon request.

## Nomenclature

ω	coefficient of maximum width
A	coefficient of maximum depth
$A_{1}$, $A_{2}$	maximum depth regulation interval
y	calculated elevation of profile (μm)
$y_{0}$	original elevation of surface (μm)
*x*	calculated position (μm)
$x_{c}$	polar transverse coordinates (μm)
Ra	surface roughness (μm)
l	calculated length (mm)
*Z*	elevation of rough peaks and valleys (μm)
$E_{d}$	laser energy density (mJ.mm^−2^)
*E*	laser energy (mJ)
*d*	spot diameter (μm)
$d_{cn}$, $d_{cp}$	width relative to the vertex of the calibrated profile (μm)
$\Delta d_{cn}$, $\Delta d_{cp}$	width increment relative to the vertex of the calibrated profile (μm)
$d_{max}$, $d_{cmax}$	maximum depth of calculated and calibrated profile (μm)
$d_{c}$	depth of calibrated profile (μm)
*D1~D5*	LSPTwC parameter of different spot diameters

## Abbreviations

LST	laser surface texturing
LSP	laser shock peening
LSPwC	laser shock peening without coating
LSPTwC	laser surface texturing by laser shock peening without coating
LOL	loss of lubrication
OM	optical microscope
SEM	scanning electron microscope
AFM	atomic force microscope
